# Promoting crystallization of intrinsic membrane proteins with conjugated micelles

**DOI:** 10.1038/s41598-020-68689-6

**Published:** 2020-07-22

**Authors:** Thien Van Truong, Mihir Ghosh, Ellen Wachtel, Noga Friedman, Kwang-Hwan Jung, Mordechai Sheves, Guy Patchornik

**Affiliations:** 1 Department of Chemical Sciences, Ariel University, 40700 Ariel, Israel; 2 Faculty of Chemistry, Weizmann Institute of Science, 76100 Rehovot, Israel; 3 Department of Life Science and Institute of Biological Interfaces, Sogang University, Seoul, 121-742 South Korea

**Keywords:** Electron microscopy, X-ray crystallography

## Abstract

A new technique for promoting nucleation and growth of membrane protein (MP) crystals from micellar environments is reported. It relies on the conjugation of micelles that sequester MPs in protein detergent complexes (PDCs). Conjugation via amphiphilic [metal:chelator] complexes presumably takes place at the micelle/water interface, thereby bringing the PDCs into proximity, promoting crystal nucleation and growth. We have successfully applied this approach to two light-driven proton pumps: bacteriorhodopsin (bR) and the recently discovered King Sejong 1–2 (KS1–2), using the amphiphilic 4,4′-dinonyl-2,2′-dipyridyl (Dinonyl) (0.7 mM) chelator in combination with Zn^2+^, Fe^2+^, or Ni^2+^ (0.1 mM). Crystal growth in the presence of the [metal–chelator] complexes leads to purple, hexagonal crystals (50–75 µm in size) of bR or pink, rectangular/square crystals (5–15 µm) of KS1–2. The effects of divalent cation identity and concentration, chelator structure and concentration, ionic strength and pH on crystal size, morphology and process kinetics, are described.

## Introduction

Membrane proteins (MPs) constitute ~ 23% of the human proteome^1^. They are responsible for active and passive transport, energy transduction and signalling, and when malfunctioning, for diverse forms of human pathology^2–5^. Membrane proteins therefore represent major targets for biochemical studies and drug development^6–9^ for which high resolution structure determination of MPs is essential.

MPs form both two dimensional (2D) and three dimensional (3D) crystals. 2D crystals are generally formed by inserting purified, protein detergent complexes (PDCs) into lipid bilayers^10^. However, 3D crystals have been the major source of high-resolution structure determination. 3D MP crystals are commonly subdivided into two groups: Type I—where MPs are organized in planar sheets, through protein–detergent–lipid hydrophobic interactions, stacked on top of one another by polar interactions; and Type II 3D crystals—generated by PDC assembly^11,12^. Recent surveys have shown that the majority of high-quality 3D crystals are Type II^12,13^. Despite recent technical advances, including: (a) overexpression of MPs in diverse hosts^14–16^; (b) development of novel detergents and lipids that improve MP extraction from their native membranes^17,18^; (c) automation and miniaturization of crystallization trials^19^ and (d) improvement of X-ray sources and reduction of radiation damage^20^, obtaining high quality, 3D single crystals of MPs nevertheless remains one of the most significant challenges facing structural biologists.

**Figure 1:**
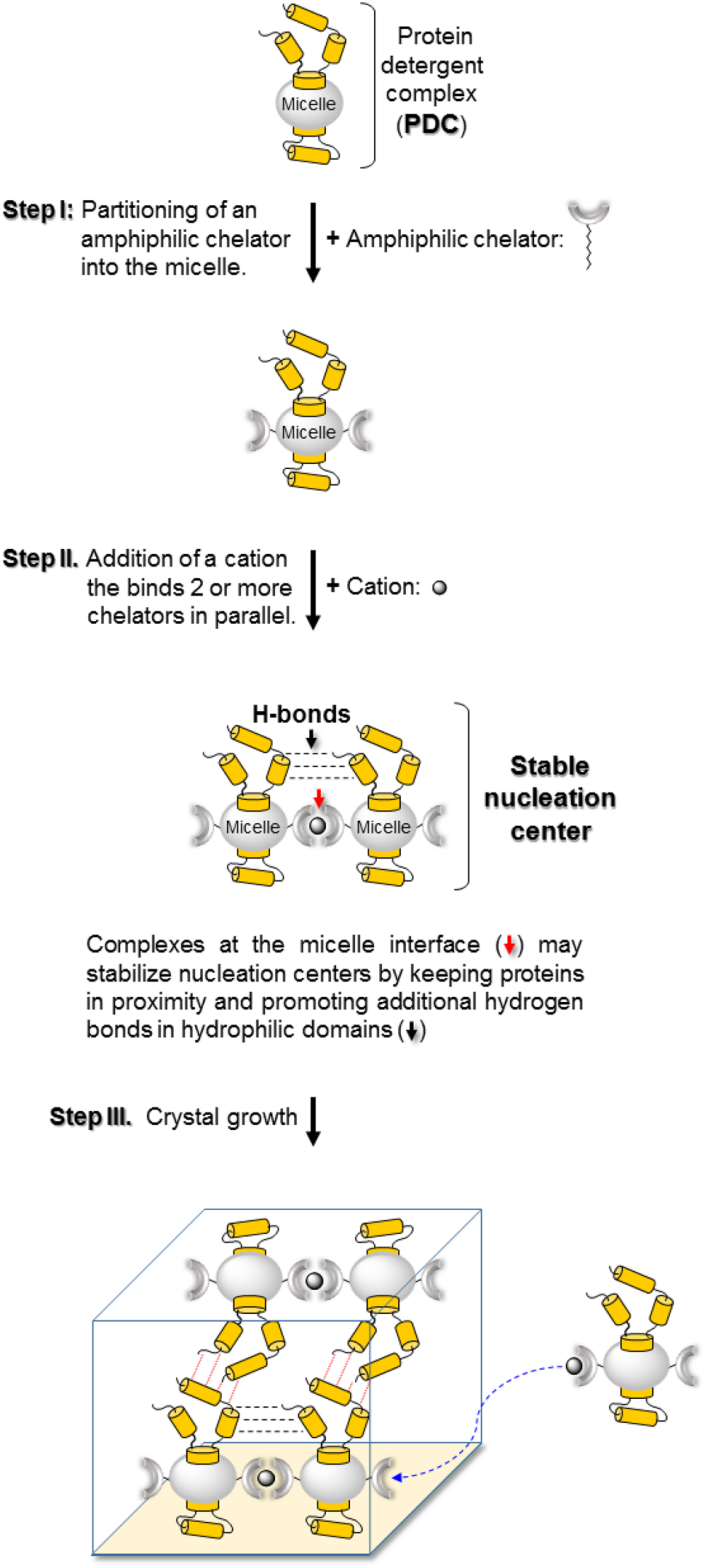
Crystallization strategy. Spontaneous partitioning of an amphiphilic chelator into membrane protein detergent complexes (PDC) (Step I). Conjugation of the PDCs is accomplished by the addition of divalent metal cations capable of binding 2–3 chelators at the micelle/water interface (Step II, red arrow) Step III—growth of three-dimensional crystals.

Since the introduction of lipidic cubic phases in 1996 by Landau and Rosenbusch^21^ no conceptually new pathway to MP crystallization from micellar environments has been introduced. In our work, we have focused on the possibility of promoting crystal nucleation and growth by exploiting the physical chemistry of the detergent component in PDCs. Until now, detergent micelles have primarily served as a shield against membrane protein aggregation in aqueous solution and/or as a stabilizing environment. However, we have found that it is possible to exploit the micellar moiety for a new objective, i.e., directing PDCs to assemble via specifically conjugated micelles under controlled conditions. Such an objective builds on recent studies that have shown how a variety of amphiphilic [metal:chelator] complexes presumably located at the micelle/water interface, are able to promote large-scale micellar conjugation^22,23^. Here we demonstrate that successful implementation of this approach on PDCs (outlined in the cartoon in Fig. 1) leads to the crystallization of the light driven proton pumps: bacteriorhodopsin (bR) and the recently discovered King Sejong 1–2 (KS1–2).

## Results

### Conjugating empty, non-ionic β-d-1-octylthioglucoside (OTG) micelles

**Figure 2:**
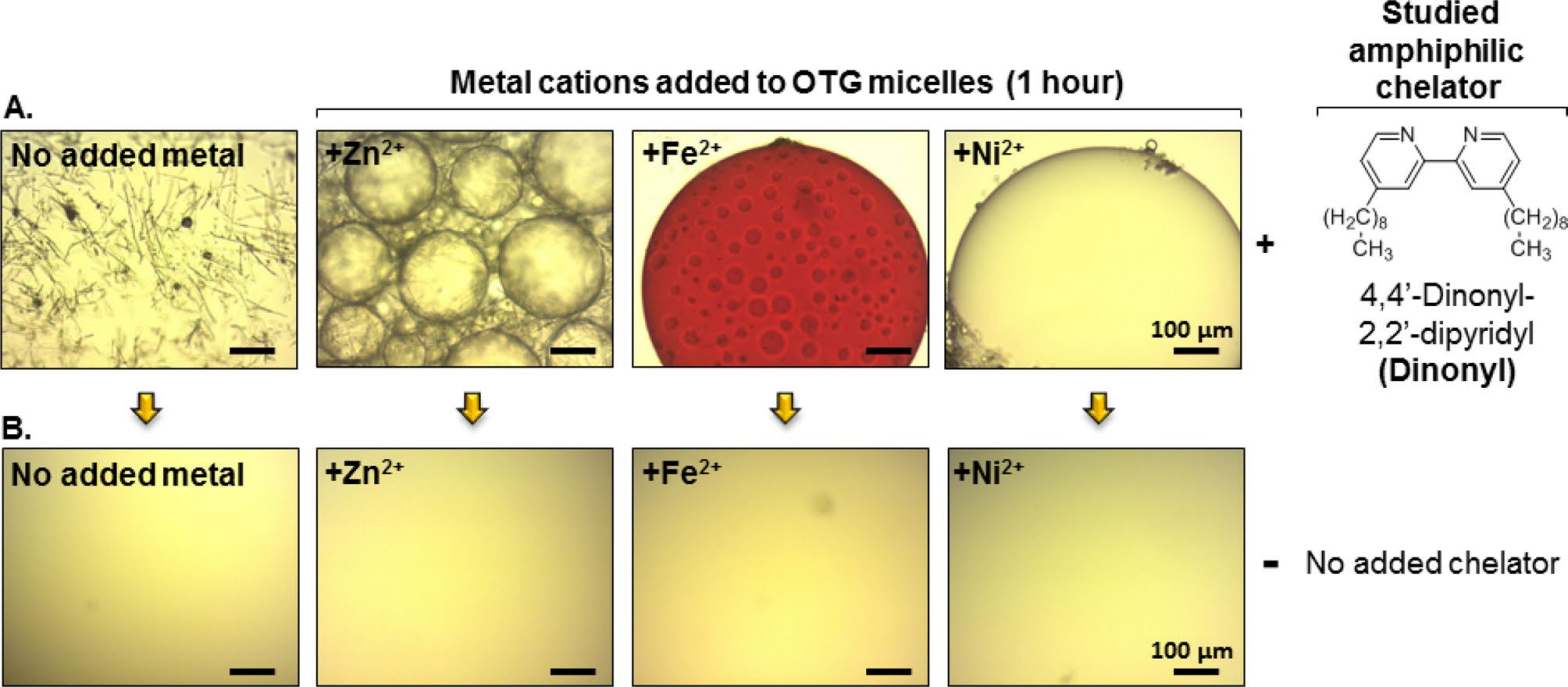
Conjugation of empty OTG micelles. **(A)** Light microscope images of hanging drops containing OTG (30 mM), the amphiphilic chelator, Dinonyl (1 mM) and cations as indicated (3 mM). After 1 h of incubation at 19 °C in the presence of 200 mM NaCl, phase separation of oil-rich globules is observed. The red color in the presence of Fe^2+^ derives from the [(Dinonyl)_3_:Fe^2+^] associate. Scale bar in all images represents 100 μm. (**B)** All conditions as in (**A**), except that no chelator is present.

Non-ionic β-d-1-thioglucoside (OTG) micelles, devoid of MPs, were conjugated via the amphiphilic chelator 4,4′-dinonyl-2,2′-dipyridyl (Dinonyl) in association with divalent metal cations: Zn^2+^, Fe^2+^, or Ni^2+^. Each cation is able to bind Dinonyl at a molar ratio of 1:3, respectively [(Dinonyl)_3_:M^2+^]^24^ (Fig. 2). Micellar conjugation was indeed found to be efficient with each of the three cations studied and led within 60 min to macroscopic phase separation in the form of oil-rich globules (Fig. 2A). In the absence of the cations, the chelators precipitated as elongated threads (Fig. 2A, left) and in the absence of the Dinonyl chelator, but retaining the same cation concentration, no phase separation was observed (Fig. 2B).

### Conjugating membrane protein-containing OTG/phospholipid mixed micelles

**Figure 3:**
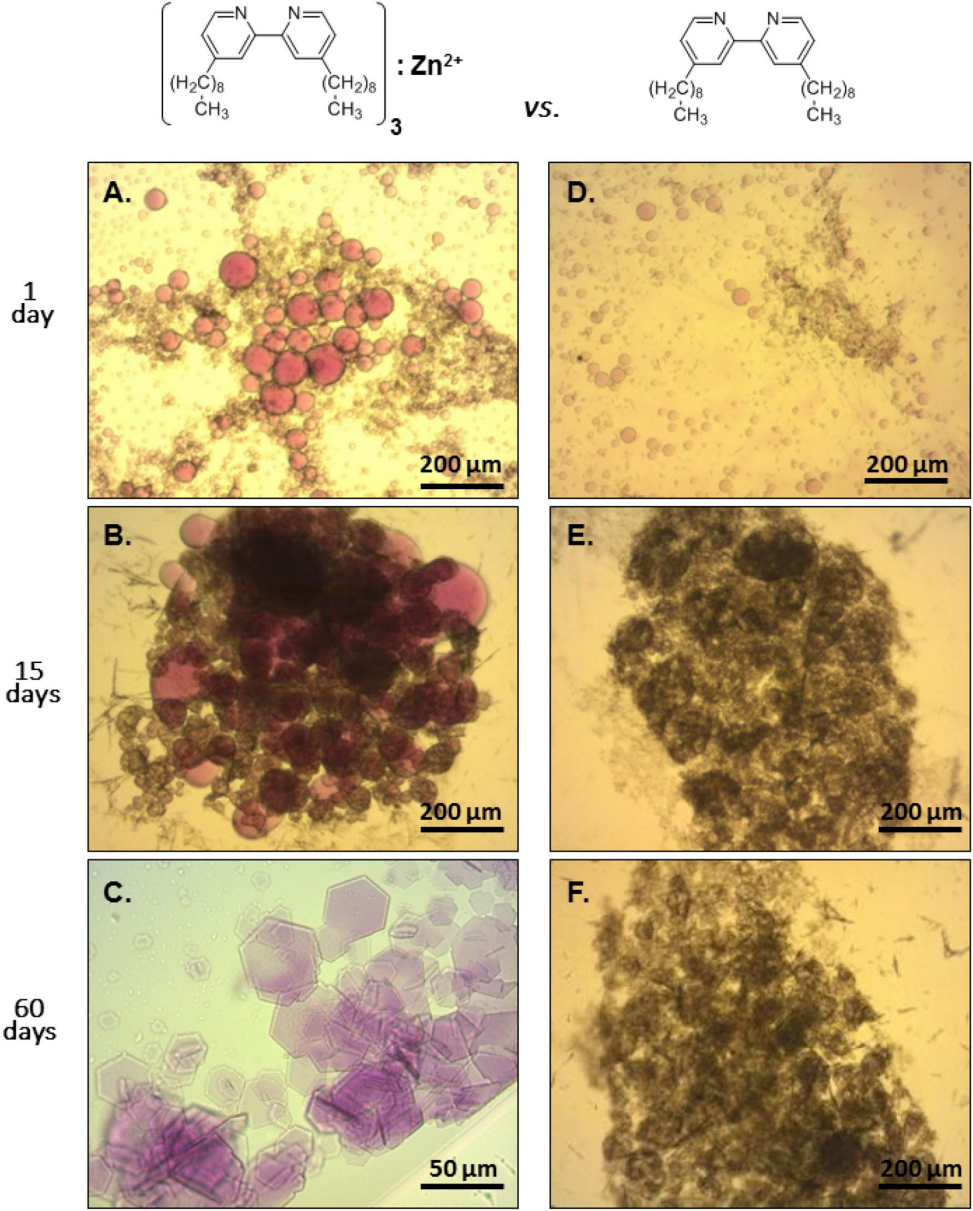
Role of the amphiphilic [(Dinonyl)_3_:Zn^2+^] complex in promoting crystallization of bR. *LHS*. Light microscopy images of hanging drops containing OTG-solubilized purple membranes (see “Methods” section) conjugated via the [(Dinonyl)_3_:Zn^2+^] couple (0.7 mM and 0.1 mM, respectively), 50 mM Na citrate (pH 5.2), 100 mM NaCl at the times indicated at 19 °C in the dark. Reservoir composition: 0.5 ml of 0.5 M ammonium sulfate. *RHS: *Hanging drops as for *LHS*, containing the amphiphilic chelator but lacking Zn^2+^ ions.

The conjugation of empty OTG micelles paved the way to assessing this approach with PDCs containing bR, native phospholipids and OTG (Fig. 3). The PDCs had been obtained by dissociating purple membranes with OTG (see “Methods” section) and validating that the characteristic bR absorption spectrum had been preserved (online Supplementary Information, Figure S1). With the availability of micellar preparations containing native bR, the effects of three divalent cations (Fe^2+^, Co^2+^, Cd^2+^) on the efficiency of conjugation were studied (online Supplementary Information, Figure S2). We found that the [(Dinonyl)_3_:Zn^2+^] complex successfully conjugated micellar aggregates and generated large purple oil-rich globules (20–100 µm) after 1 day of incubation in the dark at 19 °C (Fig. 3A). Control experiments in the absence of Zn^2+^, demonstrated the contribution of Zn^2+^ ions to the conjugation process. Without Zn^2+^, globules reached a maximal size of ~ 30 µm and were significantly less purple, indicating a lower bR concentration within the globule (Fig. 3D, upper right image). The fact that globules form upon introduction of the Dinonyl chelator, but without zinc ions, is likely due to the presence of the ammonium sulfate (AS) precipitant. The latter, like many other precipitants commonly used in macromolecular crystallization protocols *(*e.g. PEGs), compete with solutes in the system for binding to water molecules and by so doing, reduce their water-solubility. This holds true also for hydrated PDCs which, upon introduction of AS, are dehydrated and this in turn leads to their clustering and fusion into stable oil-rich, purple globules as shown in Fig. 3D.

### Growth of type II 3-dimensional bR crystals via [(Dinonyl)_3_:Zn^2+^] conjugated OTG-phospholipid mixed micelles

After 15 days incubation of the bR-containing conjugated PDCs in the dark at 19 °C, a mixture of dark precipitates combined with larger purple globules (150 µm) were visible (Fig. 3B) and after 60 days, thin, hexagonal crystals (10–50 µm) appeared (Fig. 3C). Control experiments under identical conditions but without the Dinonyl chelator demonstrated that Zn^2+^ ions (0.1 mM) on their own do not promote crystal growth (not shown).

### Growth of 3-dimensional bR crystals in [(Dinonyl)_3_:Ni^2+^] conjugated OTG/phospholipid mixed micelles

**Figure 4:**
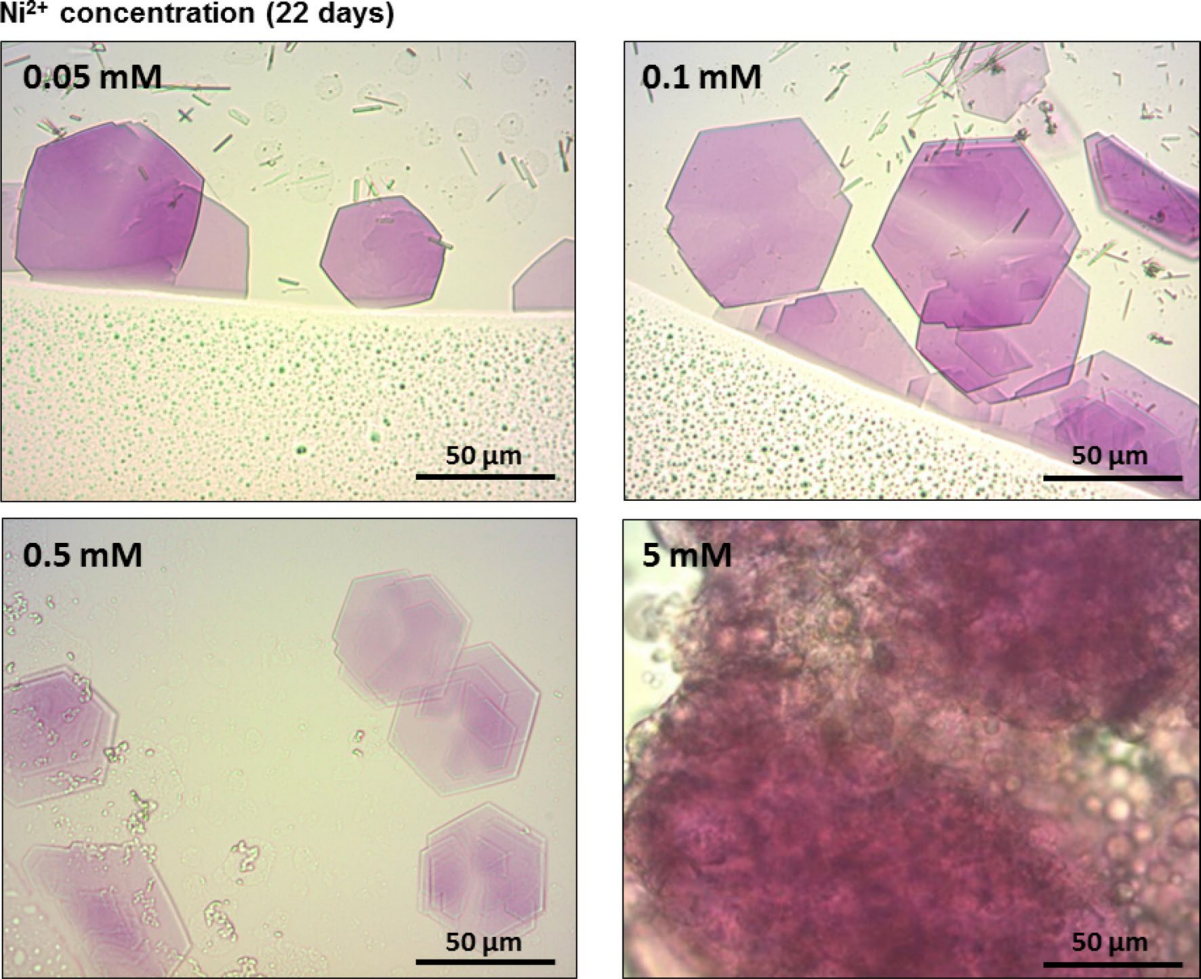
Crystallization of bR via [(Dinonyl)_3_:Ni^2+^] conjugation. Light microscope images of hanging drops containing OTG-solubilized, purple membranes (see “Methods” section), Dinonyl (0.7 mM) and Ni^2+^ concentrations as indicated with 50 mM Na citrate (pH 5.2) and 100 mM NaCl) after 22 days at 19 °C in the dark. Reservoir composition: 0.5 ml of 0.5 M ammonium sulfate.

Exchanging Ni^2+^ ions for Zn^2+^ produced similar crystal growth (Fig. 4). Hexagonal thin, purple-colored crystals reached their largest size (~ 70–75 µm) with Ni^2+^ concentration 0.05–0.1 mM (Fig. 4). However, at higher Ni^2+^ concentration (5 mM), no crystals were observed. Though a tendency to crystal stacking was observed with 0.1 mM Ni^2+^ ions as well as Zn^2+^ ions, at lower Ni^2+^ concentration (0.05 mM) this tendency was suppressed. Moreover, cation identity appeared to modify process kinetics. More rapid crystal growth was observed with Ni^2+^ ions (22 days) as compared to Zn^2+^ ions (40–60 days) under identical conditions. Attempts to crystallize bR with other cations known to bind Dinonyl at the same molar ratio (1:3, respectively^24^), *i.e.,* Fe^2+^, Co^2+^ or Cd^2+^ failed to produce any crystals.

### Chelator structure and growth of bR crystals

Other parameters that were expected to affect crystal growth included chelator structure and concentration, molarity of NaCl and pH. The chemical structure of the Dinonyl chelator is available in Fig. 3. Repetition of the crystallization protocol with a Dinonyl analog, 4,4′-diphenyl-2,2′-dipyridyl (Diphenyl), did promote crystallization of bR (Supplementary Information, Figure S3). However, the crystals were smaller (max. 20 µm) and were not hexagonal (online Supplementary Information, Figure S3). Changing the Dinonyl concentration at constant Ni^2+^ concentration (0.1 mM), revealed a dramatic effect (online Supplementary Information, Figure S4). With 0.175 mM Dinonyl, hexagonal purple crystals grew within 21 days, whereas with 1 mM Dinonyl, no crystals were observed at all, even after 60 days (Figure S4, inset). An additional important parameter was found to be NaCl concentration. The largest crystals grew in the presence of 100 mM NaCl. With 20 mM NaCl, there were few crystals and they exhibited a globular morphology. With 50 mM NaCl, numerous small (5–15 µm) crystals appeared. However, with 500 mM NaCl, crystal growth was abolished (online Supplementary Information, Figure S5). The pH (5.2) of the hanging drop was a constant: higher or lower pH values failed to promote crystallization (not shown).

### Crystallization of KS1–2

**Figure 5:**
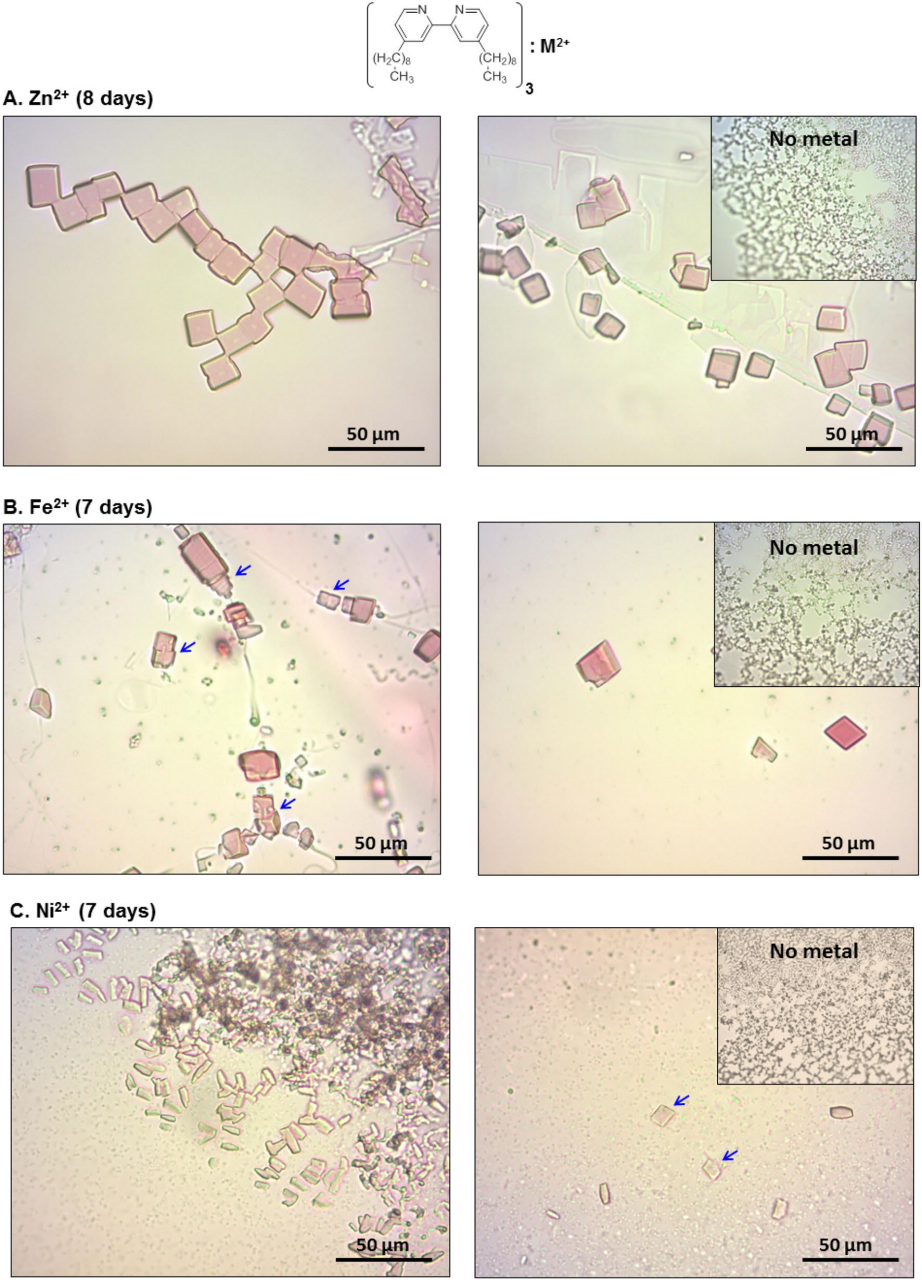
Crystals of KS1–2 obtained with the Dinonyl amphiphilic chelator (0.7 mM) and divalent metal cations (0.1 mM) as indicated. Hanging drops contain in addition: 50 mM Na citrate (pH 5.2), 100 mM NaCl and were incubated for indicated times at 19 °C in the dark. Reservoir composition: 0.5 ml of 10% (w/v) PEG-4000 in 40 mM Na citrate (pH 5.2).

Our protocol was also tested on KS1–2, a recently identified 255 amino acid membrane protein^26^ (Fig. 5). KS1–2 functions as a light-driven proton pump rhodopsin bound covalently to the retinal chromophore. Since KS1–2 pigment absorbs in the green–blue region of the visible spectrum (λ_max_ 517 nm) the proteins appear orange\purple-red in white light and pink crystals were accordingly expected. The MP was purified with DDM as the detergent (see “Methods” section) and pink rectangular/square crystals (5–15 µm) appeared only in the presence of the [(Dinonyl)_3_:Zn^2+^] complex and with the same metal (0.1 mM) and chelator (0.7 mM) concentrations used for bR (Fig. 3). After 8 days, crystals reached their maximum size. They appear to be either fused to each other (Fig. 5A, left) or independent (Fig. 5A, right) but no crystals were seen in the absence of Zn^2+^ ions (Fig. 5A, right, inset). Na citrate buffer, pH 5.2 was the same as used for bR; however, the precipitant was PEG-4000, rather than ammonium sulfate (AS). The [(Dinonyl)_3_:Fe^2+^] complex also produced fused, square pink KS1–2 crystals (Fig. 5B, left) as well as crystals with a different morphology (Fig. 5B, right). Use of the [(Dinonyl)_3_:Ni^2+^] complex led to significantly smaller, fused crystals (Fig. 5C, left), although a few diamond-shaped single crystals (5–10 µm) were also observed (Fig. 5C, right).

## Discussion

Three-dimensional, high resolution crystal structures are seriously lacking for intrinsic membrane proteins (MPs). Detergent micelle environments for MPs during crystallization trials currently represent the most popular and convenient “playground” for structural biologists; nevertheless, crystallization of MPs, particularly those with small hydrophilic loop regions, remains an unpredictable, fine art.

Two lines of evidence imply that observed crystals are comprised of the target membrane proteins: (a) No crystals of any type were observed when target membrane proteins (bR or KS1–2) were absent in the crystallization drops. (b) Interestingly, the observation of hexagonal purple bR crystals in this study was already observed with well-established crystallization strategies. Purple hexagonal bR crystals grew from lipid cubic phases^21,27^ and from the “successive fusion vesicle” strategy^28^. However, diamond shaped purple bR crystals, grew from bicelles^29^.

Investigating the possibility of bringing protein detergent complexes (PDCs) into proximity via specific micellar conjugation led to two very encouraging findings. First, crystals of target proteins (bR or KS1–2) grew only in the presence of both the chelator (Dinonyl) and appropriate metal cations. The mandatory dependence of crystal growth on these two additives is consistent with our working hypothesis, demonstrates process specificity, and the existence of a simple chemical mechanism capable of promoting crystallization. That these crystals contain native, chromophore-containing, target membrane proteins is supported by the fact that no crystals of any type were observed when MPs (bR or KS1–2) were absent from the crystallization drops. Since it is known that a change in the chromophore conformation (or its hydrolysis from the protein) leads to the loss of color, color-preservation serves as an internal indicator for the presence of the protein’s native state within the crystal. Second, once the optimal concentrations of chelator and metal had been successfully determined, crystallization was observed in > 80% of the hanging drops in each of > 10 independent trials. Such high process reproducibility may not be common in other MP crystallization protocols and demonstrates the efficiency and robust character of our approach.

Our strategy for crystallizing MPs, based on large-scale conjugation of PDCs, demonstrates several important advantages in comparison with the variety of procedures already in use by structural biologists^30–32^. These advantages include: (i) Covalent modifications, e.g. amino acid “tags”, are not required; crystallization trials of PDCs can proceed directly from optimized expression and purification of the protein under study. (ii) Concentrations of cations (0.1 mM) and amphiphilic chelator (0.7 mM), adequate for producing micelle conjugation, are sufficiently low that the native states of the MPs studied (bR and KS1–2) appear to be preserved. (iii) PDCs may contain either non-ionic detergents (e.g. DDM in the case of KS1–2) or negatively charged mixed micelles (native phospholipids + OTG) as in the case of bR. (iv) Our strategy is sufficiently flexible to permit a wide range of working conditions. The Dinonyl chelator remains functional under both acidic or basic conditions. In this study, the chelator was found to be usable after 60 days at pH 5.2. (v). The low cost and commercial availability of all components, in particular the Dinonyl chelator, facilitate rational screening of diverse MPs. The chelator and divalent metal cation are simply added to the micelle dispersion as with any other additive. Furthermore, no special instrumentation is needed. Though the crystals obtained were too small to permit structure determination by X-ray diffraction, we are encouraged by the fact that color was preserved (purple for bR and pink for KS1-2), indicating that the MP’s active site had not been distorted. Slow progress in obtaining high quality crystals of MPs remains a major bottleneck in drug design; to alleviate this problem, it will be necessary to apply as many innovative approaches as possible. We trust that our strategy will aid in overcoming this problem.

## Methods

### Materials

All-*trans* retinal, ammonium sulfate, ampicillin, DNase, isopropyl β-d-1-thiogalactopyranoside (IPTG), octyl β-d-1-thioglucoside (OTG), decyl β-d-maltoside (DM), dodecyl β-d-maltoside (DDM), ethanol, imidazole, lysozyme, MgCl_2_, 2-(*N*-morpholino) ethanesulfonic acid, (MES), NaCl, NaN_3_, Na citrate, polyethylene glycol 4000 (PEG-4000), FeSO_4_, NiBr_2_, ZnCl_2_, CoSO_4_, CdCl_2,_ 4,4′-dinonyl-2,2′-dipyridyl (Dinonyl) and 4,4′-diphenyl-2,2′-dipyridyl (Diphenyl) were obtained from Sigma-Aldrich (St. Louis, MO).

### Conjugation of octyl β-d-1-thioglucoside (OTG) micelles

Into 53 µL of DDW, 120 µL of 100 mM OTG (in DDW) and 27 µL of 15 mM Dinonyl (in EtOH) were added with vigorous vortexing. Equal volumes (e.g. 1.5 µL) of the chelator/OTG mixture and a medium containing 6 mM of either FeSO_4_, NiBr_2_ or ZnCl_2_ in 400 mM NaCl were added. Drops (e.g. 3 µL) were placed on siliconized cover-slides (VDX™ crystallization plates, Hampton Research) and incubated for 1 h at 19 °C in the dark against reservoirs containing 200 mM NaCl.

### Preparation of purple membranes

*Halobacterium salinarum* was grown from the S9 strain and purple membranes containing bacteriorhodopsin (bR) were isolated as previously described^25^.

### Expression and purification of King Sejong 1–2 KS1–2 were prepared according to Ghosh et al.^26^

Codon optimized KS1–2 was expressed in Luria–Bertani (LB) medium in *Escherichia coli* BL21 (DE3) cells. *E. coli* transformant was grown to OD (*λ*_600_) = 0.8 in the presence of ampicillin (50 μg mL^−1^) at 37 °C. The cells were then induced with 0.1% IPTG (isopropyl β-d-1-thiogalactopyranoside) and 10 μM all-*trans* retinal for 8 h. Pink-colored cells were harvested by centrifugation at 4 °C, followed by resuspension with buffer I (50 mM 2-(*N*-morpholino) ethanesulfonic acid, (MES), 300 mM NaCl, 5 mM imidazole, 5 mM MgCl_2_; pH 6) containing 1% DDM (*n*-dodecyl-β-d-maltoside) and lysed with lysozyme (0.1 mg mL^−1^) in the presence of DNase and a protease inhibitor. The mixture was stirred overnight at room temperature. The extracted protein was collected as a supernatant after centrifugation of the stirred solution at 18,000 rpm and 4 °C for 30 min. The protein was purified using a Ni^2+^ NTA histidine-tagged agarose column. The histidine-tagged protein was washed with buffer II (50 mM MES, 300 mM NaCl, 50 mM imidazole, 0.06% DDM; pH 6) and eluted with buffer III (0.06% DDM, 50 mM Tris–HCl, 300 mM NaCl, 50 mM HCl, 150 mM imidazole; pH 7.5). The eluted protein was washed and concentrated using a 0.02% DDM solution using Amicon ultra centrifugal filter devices. The final KS1-2 was kept in 0.06% DDM.

### Solubilization of purple membranes with OTG

Into 15 µL of purple membranes (OD 14) in DDW, 8.5 µL of 100 mM octylthioglucoside (OTG), 7.1 µL of 0.5 M Na citrate (pH 5.2) and 40 µL of DDW were added to a total volume of 71 µL. The system was incubated for 3 h at 19 °C in the dark, followed by a short spin (15,000 rpm, 10 min, 19 °C). The resulting transparent purple supernatant was used immediately for crystallization trials.

### Optical absorption of bR and KS1–2 with and without the [(Dinonyl)_3_:Ni^2+^] complex

The absorption spectrum of bR extracted from its native purple membrane with OTG (as described above) was measured (Figure S6, A). The characteristic absorption of native bR at 565–568 nm was preserved when the amphiphilic chelator (Dinonyl), the metal (Ni^2+^) or both were added and incubated for up to 24 h at 19 °C in the dark (Figure S2, A). However, a decrease of ~ 20% in the OD of bR at 565–568 nm was observed after 1 day of incubation in the presence of the [(Dinonyl)_3_:Ni^2+^] amphiphilic complex, presumably due to the ability of the latter to trigger bR aggregation. A similar decrease in the OD at 517 nm of KS1-2 in DDM micelles was observed in the presence of the same amphiphilic complex, after 1 day of incubation at 19 °C in the dark (Figure S7, A). However, in this case a shoulder at ~ 476 nm was also observed after 8 h of incubation which did not further change following over-night incubation at 19 °C in the dark (Figure S7, A).

### Dynamic light scattering (DLS) of bR and KS1–2 PDC’s

Samples containing either: (i) OTG (12 mM); (ii) bR (0.5–1.0 mg/mL) in OTG (12 mM) or (iii) as in (ii), but with the addition of Dinonyl (0.175 mM) and Ni^2+^ (0.1 mM) (i.e. the [(Dinonyl)_3_:Ni^2+^] amphiphilic chelating complex), were prepared and the intensity-weighted size distributions of PDC’s were determined following 10 min of incubation at room temperature using the auto correlation protocol of the Nanophox instrument (Sympatec GmbH, Germany). An identical analysis protocol was used for samples containing KS1–2 (0.5–1 mg/mL) in DDM (0.06% w\w) with or without the [(Dinonyl)_3_:Ni^2+^] amphiphilic complex. The size distributions are shown in Supplementary Figures S6-B and S7-B.

### Crystallization of bacteriorhodopsin (bR)

bR was crystallized by the hanging drop method. Into aliquots of 60 µL of freshly prepared OTG solublilized purple membranes, 3 µL of 15 mM Dinonyl (or Diphenyl) in ethanol were added with vigorous vortexing. This medium, containing bR, native phospholipids, OTG and the amphiphilic chelator, was further incubated for 15 min (at 19 °C in the dark) prior to use. Hanging drops (4 µL) contained 2 µL of the protein detergent complex (PDC) preparation and 2 µL containing 0.25–0.75 M ammonium sulfate (AS), 0.1 or 0.5 mM ZnCl_2_ (or NiBr_2_), 0–500 mM NaCl, 25–75 mM Na Citrate (pH 5.2) all in DDW and 2.5% ethanol. Drops (4 µL) were placed on siliconized cover-slides (VDX™ crystallization plates, Hampton Research) and incubated at 19 °C in the dark against reservoirs containing 0.5 mL 2–2.5 M AS.

### Crystallization of KS1–2

KS1–2 was crystallized by the hanging drop method using a protocol similar to that applied in the case of bR. Into aliquots of 60 µL KS1-2 (OD 3.7) in 0.06% dodecyl maltoside (DDM) and 0.04% NaN_3_, 3 µL of 15 mM Dinonyl in ethanol were added with vigorous vortexing and incubated for 15 min at 19 °C in the dark. Hanging drops (3 µL) contained 1.5 µL of the KS1–2/DDM/Dinonyl preparation and 1.5 µL containing 5% (w/v) PEG-4000, 0.5 mM ZnCl_2_, NiBr_2_ or FeSO_4_ in 80 mM Na citrate (pH 5.2). Drops were placed on siliconized cover-slides (VDX™ crystallization plates, Hampton Research) and incubated at 19 °C in the dark against reservoirs (0.5 mL) containing 10% (w/v) PEG-4000 in 40 mM Na citrate (pH 5.2).

### Light microscopy

Images were obtained using an Olympus CX-40 light microscope equipped with an Olympus U-TV1X-2 digital camera.

### UV–visible spectroscopy

Absorption measurements were performed using the HP 8453 UV–Vis spectrophotometer.

## Supplementary information

Supplementary Information. (DOCX 2737 kb)
