# Supplementary material for: Promoting crystallization of intrinsic membrane proteins with conjugated micelles
Source: Sci Rep. 2020 Jul 22;10:12199. doi: 10.1038/s41598-020-68689-6 (PMC7376161; doi:10.1038/s41598-020-68689-6)
Supplement: Supplementary file 1 — Supplementary Information. (DOCX 2737 kb) [file 41598_2020_68689_MOESM1_ESM.docx]

**Promoting Crystallization of Intrinsic Membrane Proteins with Conjugated Micelles**

**Supporting information**

Thien Van Truong ^1^, Mihir Ghosh ^2^ Ellen Wachtel ^2^, Noga Friedman ^2^, Kwang-Hwan Jung ^3^, Mordechai Sheves ^2^ and Guy Patchornik ^1^*****

^1^ Department of Chemical Sciences, Ariel University, 40700, Israel.

^2^ Faculty of Chemistry, Weizmann Institute of Science, 76100, Rehovot, Israel.

^3^ Department of Life Science and Institute of Biological Interfaces, Sogang

University, Seoul 121-742, South Korea.

*Corresponding author:

Email: [guyp@ariel.ac.il](mailto:guyp@ariel.ac.il)

**
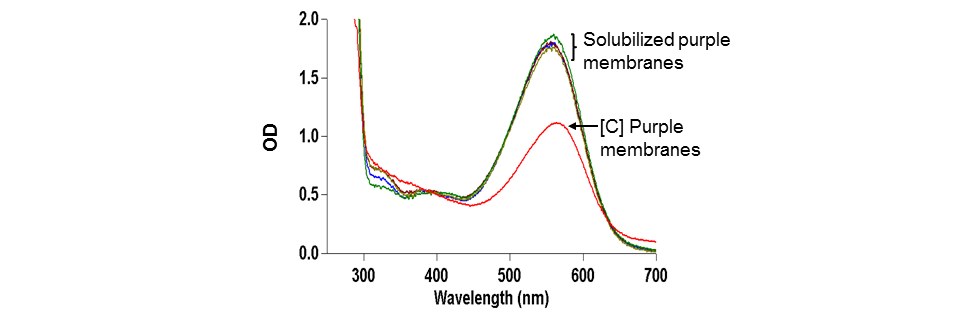
**

**Figure S1: UV absorption of purple membranes solubilized with OTG. A.** The UV absorption of freshly prepared solubilized purple membranes (see the online Methods section) was measured as a function of time: 3 hours - 560 nm (green line); 1 day - 558 nm (brown line); 4 days - 558 nm (olive line); 7 days - 558 nm (blue line); Control [C]- native purple membranes (0.5 mg \ mL) not subjected to solubilization - 562 nm (red line).


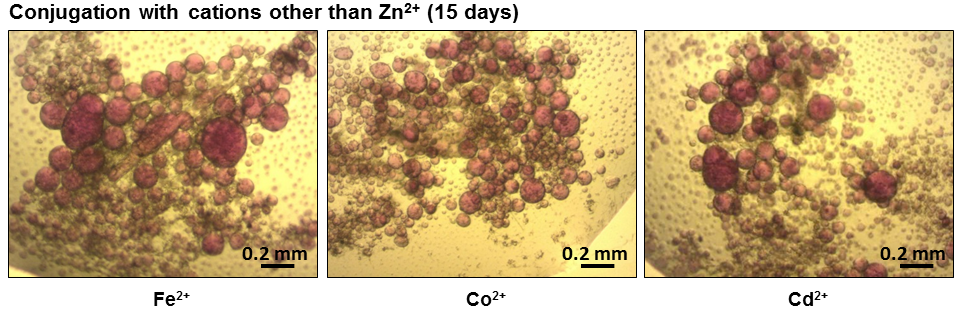


**Figure S2:** Light microscope images of conjugated protein-detergent-lipid complexes (PDCs) containing bR (see the online Methods section) incubated for 15 days at 19 °C in the dark with the amphiphilic Dinonyl chelator (0.7 mM) and cations as indicated (0.1 mM) under conditions identical to those used with Zn^2+^ ions . Hanging drop composition: 50 mM Na citrate (pH 5.2), 100 mM NaCl, 0.25 M ammonium sulfate. Reservoir composition: 0.5 M ammonium sulfate.


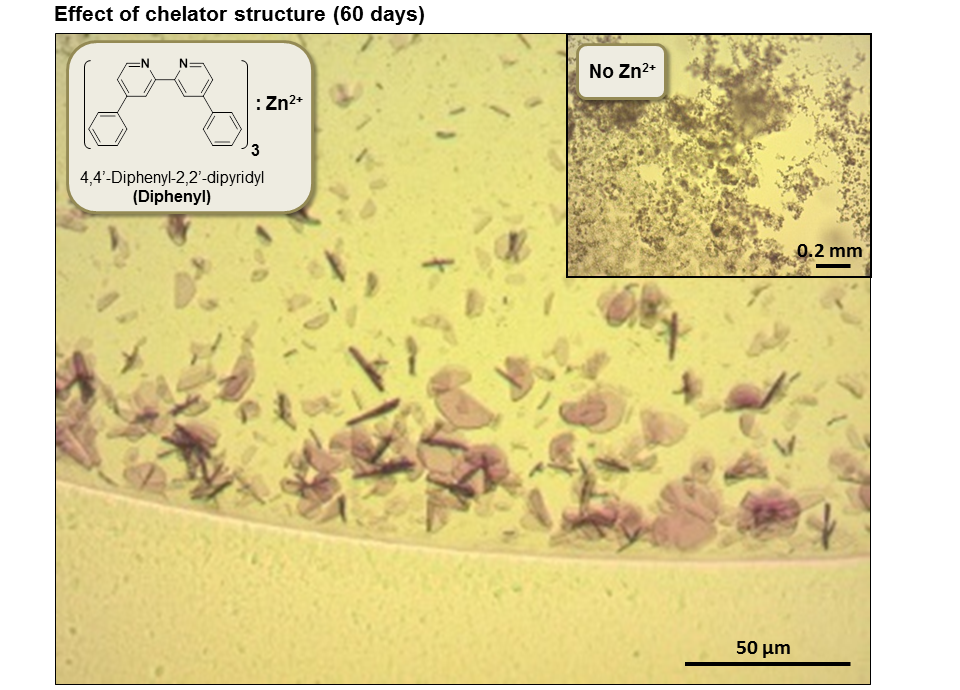


**Figure S3: Light microscope images of bR crystals obtained with [Diphenyl)_3_:Zn^2+^] conjugated PDC’s.** Freshly prepared and conjugated OTG-solubilized purple membranes (see the online Methods section) gave rise to purple-colored crystals under the same conditions used with the Dinonyl chelator (see the caption to Figure S2).


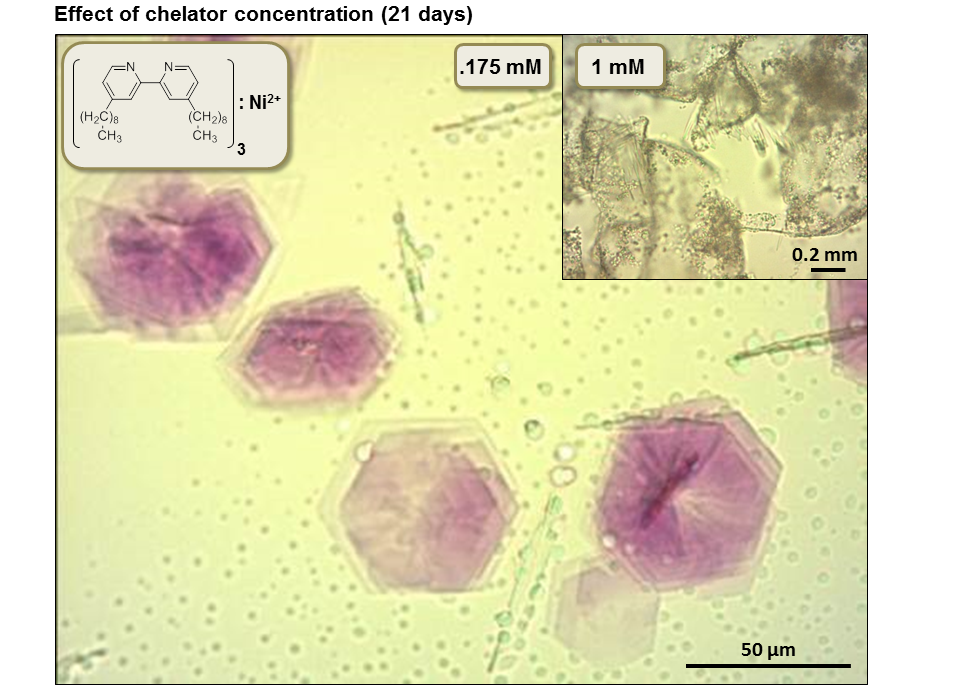


**Figure S4: bR crystals obtained from [(Dinonyl)_3_:Ni^2+^]-conjugated mixed OTG/phospholipid micelles** (Drop composition: 50 mM Na citrate (pH 5.2), 0.25 M ammonium sulfate, 0.1 mM Ni^2+^, 100 mM NaCl, 19 °C; Reservoir composition - 2 M ammonium sulfate (see the online Methods section).


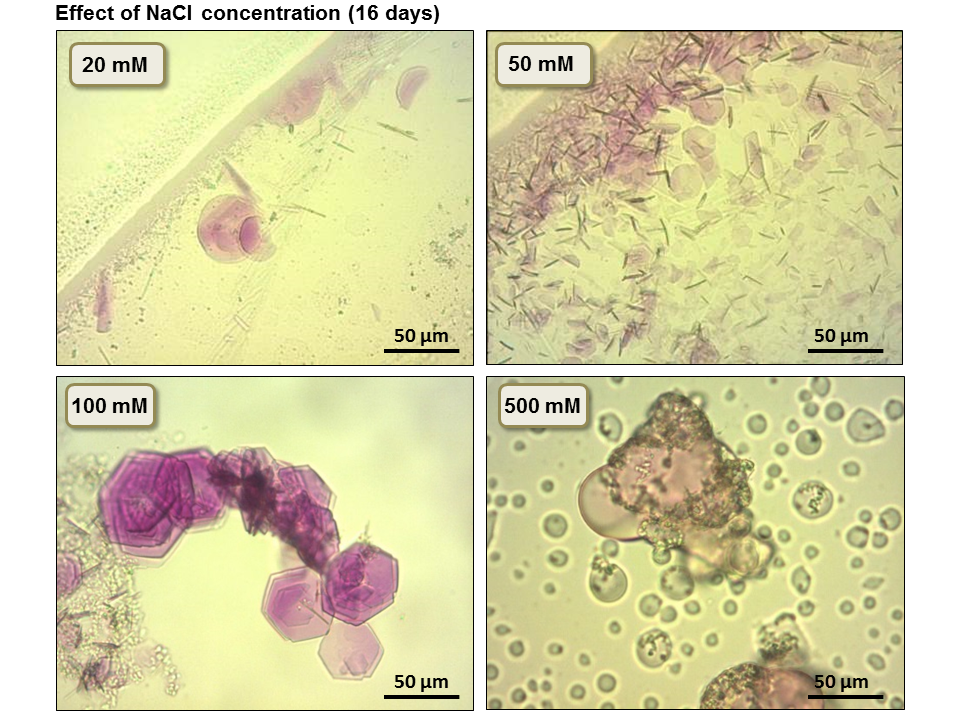


**Figure S5: bR crystals obtained with [(Dinonyl)_3_:Ni^2+^]-conjugated mixed micelles** (Dinonyl 0.7 mM, 0.1 mM Ni^2+^). Hanging drop composition: 25 mM Na citrate (pH 5.2), 0.25 M ammonium sulfate, 0.1 mM Ni^2+^, 19 °C; Reservoir composition 2 M ammonium sulfate. NaCl concentrations as indicated. (see the online Methods section).


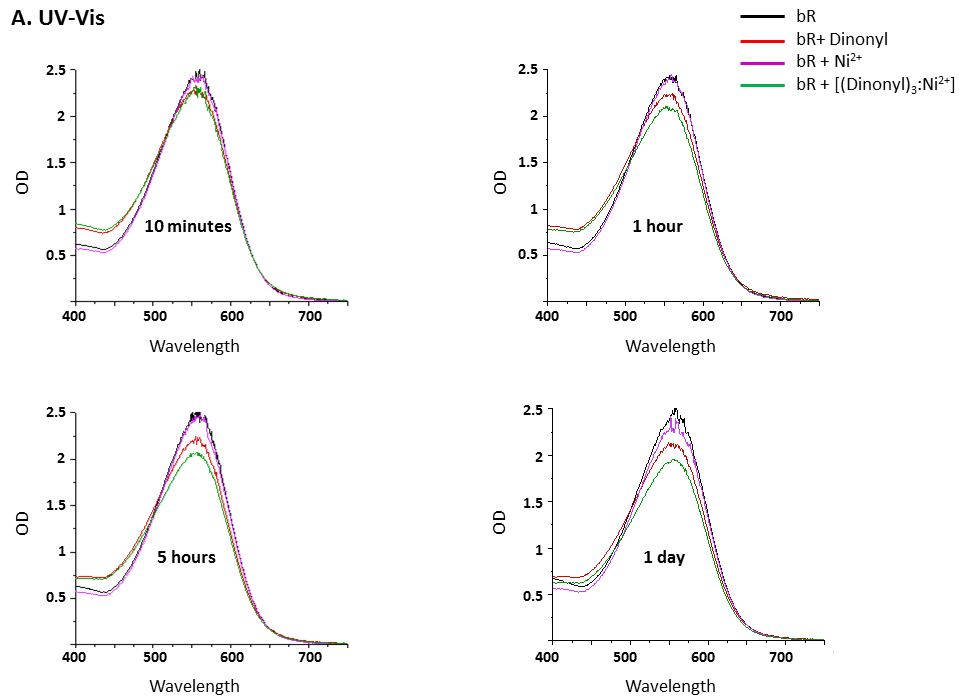


**
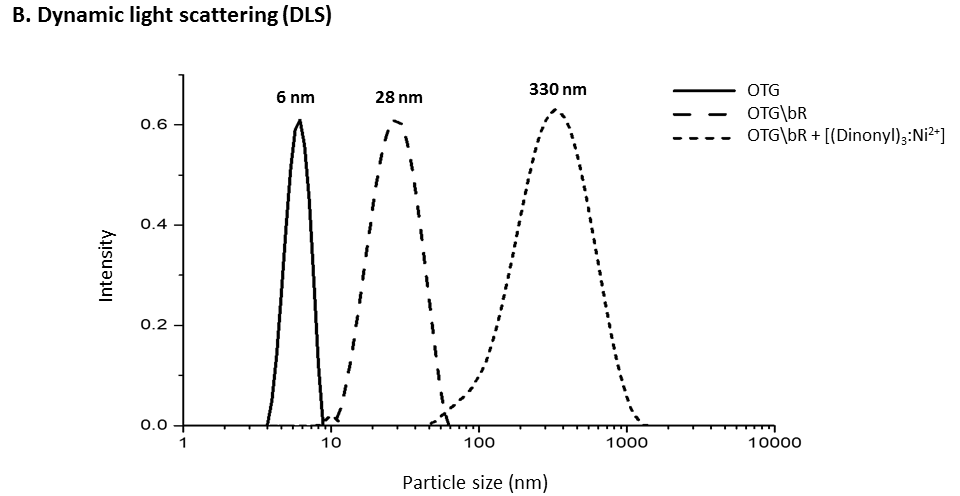
**

**Figure S6: A. Effect of Dinonyl, Ni^2+^ and the [(Dinonyl)_3_:Ni^2+^] amphiphilic complex on the optical absorption of bR.** Samples containing bR solubilized in OTG (as described in the on-line Methods section) were subjected to UV-Vis spectroscopic analysis in the absence or presence of the indicated additives at indicated time points. **B. DLS analysis.** DLS measurement of the hydrodynamic particle size of OTG micelles (^_____^); of the OTG / bR PDC (-- --); and of the OTG / bR sample 10 minutes after addition of the [(Dinonyl)_3_:Ni^2+^] amphiphilic chelating complex (- - -).


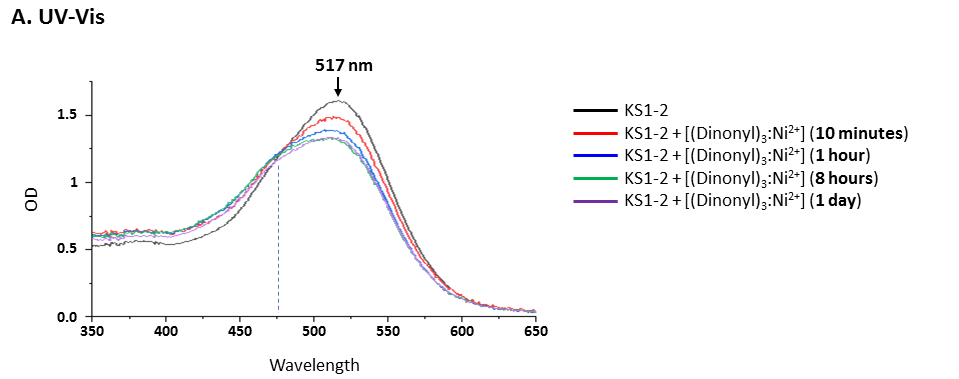


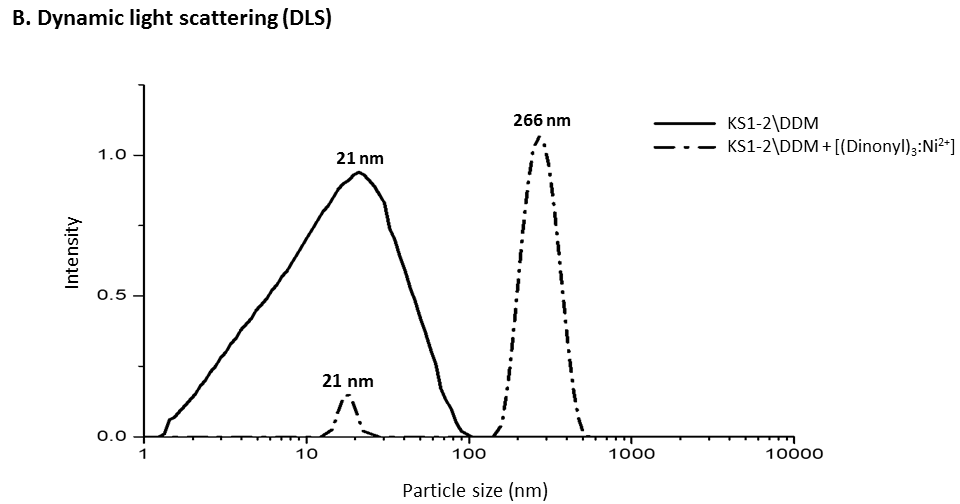


**Figure S7: A. Effect of the [(Dinonyl)_3_:Ni^2+^] amphiphilic complex on KS1-2 optical absorption.** Samples containing solubilized KS1-2 in DDM (as described in the on-line Methods section) were subjected to UV-Vis spectroscopic analysis in the absence or presence of the [(Dinonyl)_3_:Ni^2+^] amphiphilic complex at the indicated time points. **B. DLS analysis.** DLS measurements of the hydrodynamic particle size of KS1-2/DDM (^____^) and KS1-2/DDM 10 minutes after addition of the [(Dinonyl)_3_:Ni^2+^] amphiphilic chelating complex(- • -).
